# Microbiome-orchestrated cross-organ immunity in autoimmunity: from metabolites to therapeutic targets

**DOI:** 10.3389/fimmu.2026.1761834

**Published:** 2026-05-25

**Authors:** Xianlin Rao, Li Zou, Xiaoyu Cai, Yao Yao, Lingling Zhong

**Affiliations:** 1 Department of Infectious Disease, Tongde Hospital of Zhejiang Province, Hangzhou, Zhejiang, China; 2 Department of Pharmacy, Tongde Hospital of Zhejiang Province, Hangzhou, Zhejiang, China; 3 Department of Pharmacy, Affiliated Hangzhou First People’s Hospital, School of Medicine, Westlake University, Hangzhou, China; 4 Department of Pharmacy, Women’s Hospital School of Medicine, Zhejiang University, Hangzhou, China

**Keywords:** autoimmunity, cross-organ immunity, gut microbiota, gut–organ axis, microbial metabolites, precision therapeutics

## Abstract

Autoimmune diseases are systemic disorders in which barrier-site immune activation, especially in the gut, can reshape inflammatory programs in distant organs. This review advances a metabolite-centered, cross-organ framework for understanding how gut microbial ecology influences autoimmunity beyond individual gut-organ axes. We synthesize evidence that short-chain fatty acids, bile acid derivatives, tryptophan catabolites, polyamines and related microbial products act as mobile biochemical checkpoints linking intestinal barrier integrity, pattern-recognition signaling, immune-cell metabolism and tissue-specific inflammation in joints, kidneys, skin, lungs and the central nervous system. Across these axes, shared mechanisms include barrier failure, altered microbial metabolite pools, dysregulated MAMP sensing, trafficking or systemic conditioning of lymphoid and myeloid cells, and local stromal imprinting in target organs. We also discuss sex-dependent microbiome-immune interactions, including the microgenderome concept, as a framework for explaining why microbiome composition, hormone metabolism and immune responses may shape autoimmune risk and treatment response differently in females and males. Finally, we evaluate multi-omics, single-cell and spatial profiling, organ-on-chip platforms and causal computational tools, and we outline translational strategies ranging from diet, probiotics, fecal microbiota transplantation and engineered consortia to pharmacologic targeting of metabolite receptors. By treating microbial metabolites as actionable cross-organ immune checkpoints, this review highlights opportunities and limitations for biomarker-guided, metabolite-focused precision therapy in autoimmunity.

## 1 Introduction

Autoimmune diseases such as rheumatoid arthritis, systemic lupus erythematosus, inflammatory bowel disease, multiple sclerosis and immune-mediated kidney and skin diseases are increasingly recognized as systemic disorders rather than conditions confined to a single target organ. Persistent loss of self-tolerance, chronic innate and adaptive immune activation and tissue inflammation converge to produce cross-organ involvement affecting synovial joints, glomeruli, skin, the central nervous system and lung parenchyma (1, 2).

The intestinal mucosa has emerged as a critical upstream site where systemic autoimmunity can be initiated or amplified. In rheumatoid arthritis, mucosal immune activation and altered intestinal communities have been linked to anticitrullinated protein antibody production and preclinical disease risk, while expansion of Prevotella copri and transfer of dysbiotic microbiota can enhance arthritis-related immune responses in experimental systems (3, 31, 34, 36). In systemic lupus erythematosus, intestinal dysbiosis, barrier damage and pathobiont translocation have been associated with systemic immune activation and lupus nephritis in patients and lupus-prone models (4, 39, 55, 115). Similar observations in multiple sclerosis and other immune-mediated diseases support a model in which the gut environment contributes to systemic immune circuits rather than simply reflecting downstream inflammation (52, 53, 116).

Microbiota-derived metabolites provide a unifying biochemical link between intestinal ecology and extraintestinal immune pathology. Short-chain fatty acids, tryptophan-derived indoles, secondary bile acids, polyamines and related small molecules can enter the circulation, engage G protein-coupled receptors, nuclear receptors and epigenetic enzymes, and reprogram T cells, B cells, innate lymphoid cells and myeloid populations in distant tissues (5, 10, 21, 24, 114). These effects are not uniform across diseases: the same metabolite pathway may be tolerogenic in one context and neutral or pathogenic in another, depending on host genetics, barrier integrity, microbial community structure and the target-organ microenvironment.

Accordingly, the field is shifting from a gut-local view of host-microbiota interactions to a cross-organ framework that links intestinal barrier disruption, microbial metabolite remodeling, immune-cell trafficking and tissue-specific stromal imprinting. Existing reviews have summarized gut dysbiosis in individual autoimmune diseases or catalogued microbiota-derived metabolites. This review differs by integrating these strands into a metabolite-centered model of microbiome-orchestrated cross-organ immunity, explicitly comparing gut-joint, gut-kidney, gut-skin, gut-lung and gut-brain axes and distinguishing associative human observations from mechanistically supported experimental evidence.

This metabolite-centered framework also has therapeutic implications. Dietary modulation, prebiotics, probiotics, postbiotics, fecal microbiota transplantation, engineered consortia and small molecules targeting metabolite receptors are being explored in autoimmune and inflammatory diseases, but translation is constrained by heterogeneity, limited causal validation, safety concerns and insufficient sex-aware trial design (85, 88, 93, 108). We therefore focus on how microbial metabolites organize cross-organ immune networks, how emerging technologies can test causality, and how biomarker-guided interventions might move the field toward precision metabolite-based therapy. 

## 2 Conceptual framework: microbiome-orchestrated cross-organ immunity

The term microbiome-orchestrated cross-organ immunity is used here as an integrative synthesis rather than as a wholly new biological entity. It brings together established concepts of mucosal immune priming, microbial pattern recognition, metabolite signaling, leukocyte trafficking and tissue-specific inflammation into a single framework for autoimmune disease. Within this framework, microbiota-derived molecular patterns and metabolites condition immune cells in the gut, imprint migratory lymphoid and myeloid populations, and reshape metabolic circuits that subsequently operate in joints, kidneys, skin, lungs and the central nervous system. Metabolites are prioritized in this review because they are measurable, diffusible, biologically active across tissues and potentially druggable, making them practical connectors between intestinal ecology and systemic autoimmunity (6, 7).

### 2.1 Defining cross-organ immunity in autoimmunity

Cross-organ immunity refers to immune communication between anatomically distinct tissues through circulating leukocytes, soluble mediators and neuroendocrine signals. Some elements are well established, including gut-dependent Treg and Th17 differentiation, systemic dissemination of microbial products after barrier disruption and the capacity of fecal microbiota transfer to alter disease phenotypes in gnotobiotic models (7, 8, 14, 15). Other aspects remain emerging, particularly the extent to which immune cells educated in the gut directly seed human target tissues and whether organ-to-gut feedback occurs in every autoimmune disease. Thus, bidirectionality should be viewed as context-dependent rather than universal. In many settings the gut functions as a priming hub, whereas in others inflamed organs may secondarily reshape systemic metabolism, medication exposure and intestinal ecology.

### 2.2 Routes of microbiome–host communication

**Figure 1:**
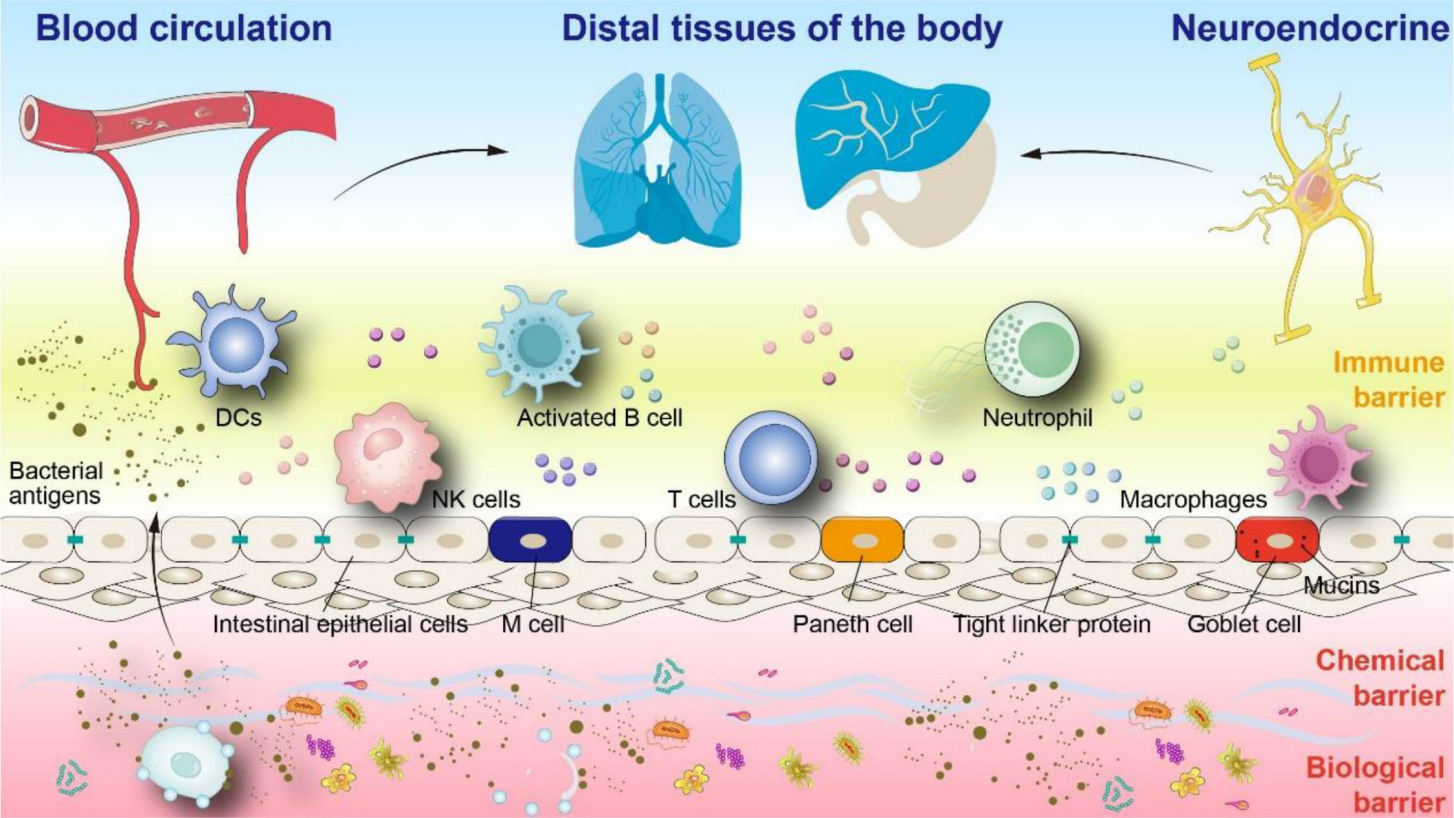
Barrier functions of the gut.

Microbiome-host communication operates through parallel and interacting routes rather than a strict hierarchy. Microbial-associated molecular patterns such as lipopolysaccharide, peptidoglycan, flagellin and muramyl dipeptide engage Toll-like and NOD-like receptors on epithelial, myeloid and lymphoid cells, shaping cytokine and costimulatory signals (6, 9). Barrier defects determine the degree to which these microbial products enter tissue and blood, thereby amplifying systemic immune activation (7, 12). **Figure 1** summarizes the multilayered gut barrier, including mucus, epithelial tight junctions, antimicrobial peptides, and immune surveillance, as the first checkpoint controlling microbial product translocation and downstream systemic immune activation. In parallel, short-chain fatty acids, secondary bile acids and tryptophan catabolites diffuse or are transported into the circulation and signal through G protein-coupled receptors, nuclear receptors and epigenetic enzymes in distant organs (10, 11). Neural and endocrine pathways, including vagal and stress-hormone circuits, add another layer of communication (13). Metabolites are emphasized here not because they are the only route, but because they integrate microbial function, diet, barrier status and host immune metabolism in a form that can be quantified and therapeutically modulated.

### 2.3 Immune circuits shaped by the microbiome

**Figure 2:**
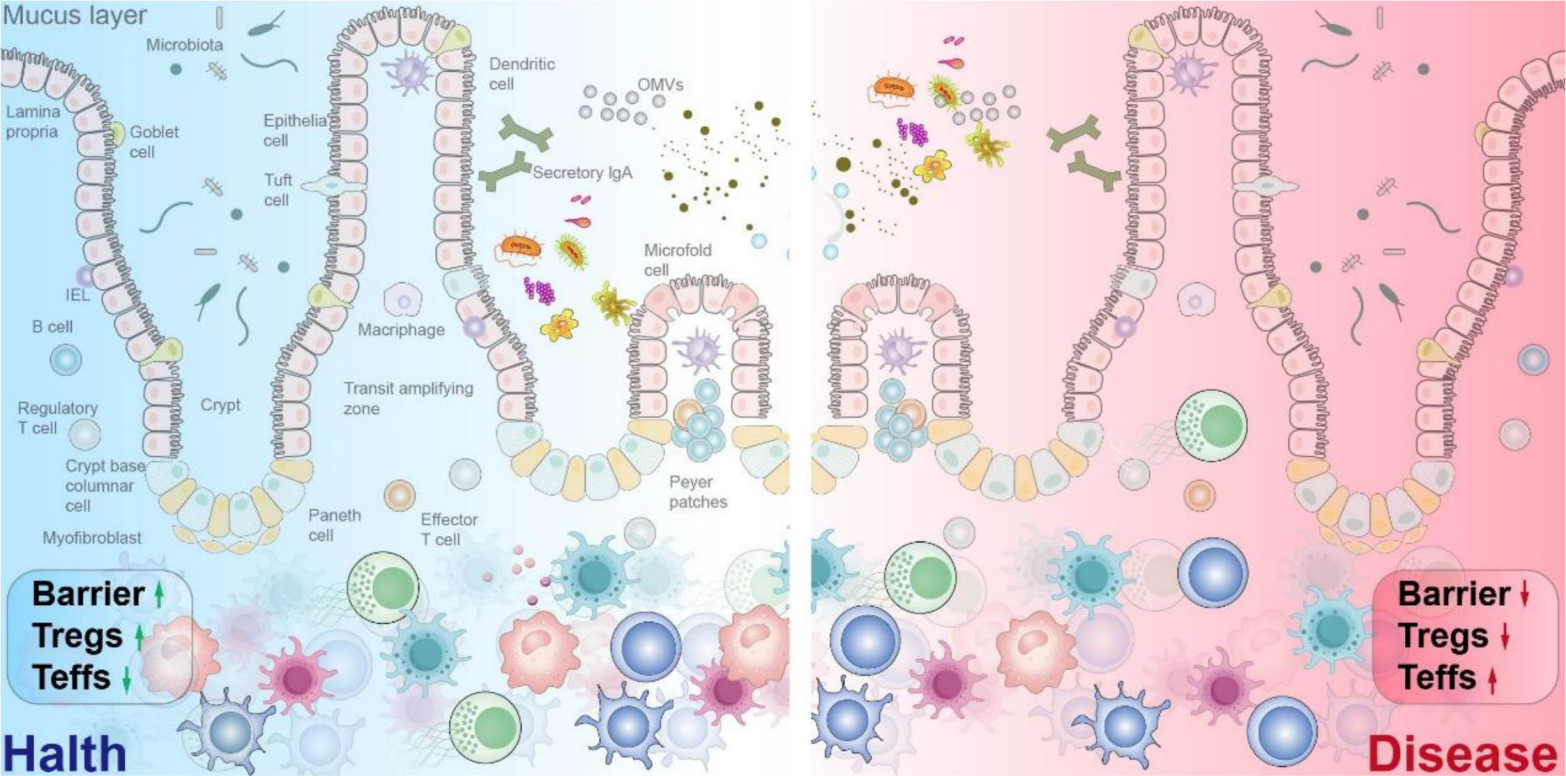
Interactions between the gut microbiota and the immune system in health and disease.

The microbiome influences immune circuits that are broadly relevant to autoimmunity, although their importance differs by disease and target organ. At the adaptive level, commensal-derived metabolites promote peripheral Foxp3+ regulatory T-cell generation and maintain colonic Treg homeostasis (14, 15). Short-chain fatty acids can shift the Treg/Th17 balance through histone deacetylase inhibition, GPR43/GPR109A signaling and changes in cellular metabolism (10, 11). Microbiota also calibrate Tfh-B cell interactions in Peyer’s patches and mesenteric lymph nodes, shaping IgA repertoires and systemic antibody responses (6). Innate lymphoid cells, particularly ILC3, respond to microbial and dietary cues and regulate tissue protection, type 3 inflammation and RORgammat+ Treg selection (16, 17). These circuits provide a common immunological vocabulary across autoimmune diseases, but their pathogenic weight varies across lupus nephritis, arthritis, psoriasis, multiple sclerosis and lung inflammation. **Figure 2** provides an overview of how gut microbial communities interact with epithelial, innate and adaptive immune cells to maintain tolerance in health and to drive dysregulated inflammatory circuits in autoimmunity.

### 2.4 Systems-level view: the microbiome–immune–metabolism triangle

The preceding routes and immune circuits converge on a microbiome-immune-metabolism triangle. This concept extends beyond descriptive multi-omics by proposing a testable architecture: microbial genes generate or transform metabolites, immune cells sense these molecules through receptors and metabolic enzymes, and host inflammatory states feedback to restructure microbial ecology and metabolite availability. Large human consortia and integrative studies show that longitudinal microbial variation is coupled to host transcriptomic, epigenomic and metabolomic states in chronic inflammatory conditions (18, 19). In autoimmunity, early multi-omics studies reveal coordinated shifts in microbial metabolic pathways, circulating metabolites and immune-cell phenotypes in lupus, rheumatoid arthritis and multiple sclerosis (7, 8, 76). The triangle therefore serves as a bridge from the cellular circuits described above to the metabolite classes discussed below, emphasizing causal modules rather than isolated associations.

## 3 Microbial metabolites as central mediators in autoimmunity

Microbiota-derived metabolites form a biochemical interface linking diet, microbial function and host immunity. Short-chain fatty acids, bile acid derivatives, tryptophan catabolites, polyamines, vitamins and other small molecules are produced in the gut lumen, enter portal or systemic circulation and reach immune niches in distant organs. Their alterations are not uniformly reproduced across all autoimmune diseases. Some patterns, such as loss of SCFA-producing capacity or altered tryptophan metabolism, recur across several cohorts, whereas others are disease-, population- or treatment-specific. Therefore, this section distinguishes clinical associations from mechanisms validated by metabolite supplementation, receptor perturbation, gnotobiotic transfer or immune-cell assays. This distinction is essential for avoiding overinterpretation while retaining the central premise that metabolite pathways are actionable nodes in cross-organ immunity (5, 20).

### 3.1 Short chain fatty acids

SCFAs such as acetate, propionate and butyrate are generated by microbial fermentation of dietary fiber and resistant starch. They reach millimolar concentrations in the colon, undergo hepatic metabolism and circulate at lower concentrations to peripheral tissues such as bone marrow, lung and brain (5). SCFAs signal through GPR43 (FFAR2), GPR41 (FFAR3), GPR109A and related receptors, and propionate and butyrate can inhibit histone deacetylases to remodel chromatin accessibility (10). Mechanistically, butyrate promotes Foxp3 induction and colonic Treg expansion while limiting Th17 polarization through HDAC inhibition and enhanced acetylation at the Foxp3 locus (10, 14, 112). These tolerogenic effects are supported by strong experimental evidence, but human data do not consistently show reduced SCFAs in every autoimmune disease or cohort. Moreover, SCFAs can enhance B-cell metabolism, class switching and antibody production; in antibody-driven settings such as lupus, this may be beneficial, neutral or potentially pathogenic depending on disease stage and cellular context (10). Thus, SCFAs should be interpreted as context-dependent immune modulators rather than uniformly anti-inflammatory metabolites.

### 3.2 Bile acid derivatives

Primary bile acids synthesized from cholesterol in the liver are conjugated and secreted into the intestine, where microbial deconjugation and transformation generate secondary bile acids. These molecules signal through FXR, vitamin D receptor and TGR5 in hepatocytes, epithelial cells and immune populations (5). The best-established immune mechanism is lineage regulation: defined bile acid derivatives such as 3-oxo-lithocholic acid and isoallo-lithocholic acid can suppress Th17 differentiation or promote peripheral Treg generation in experimental systems (21). By contrast, the causal role of altered bile acid signaling in extraintestinal autoimmune contexts remains less certain. In inflammatory bowel disease and liver-related immune disorders, dysbiosis is associated with impaired regulatory bile acid pools and skewed Th17/Tfh responses, while in multiple sclerosis altered bile acid profiles and receptor expression correlate with CNS inflammation and experimental modulation affects microglia, astrocytes and demyelination (22). These findings support bile acids as mechanistic candidates, but human evidence outside gut and liver disease remains partly correlative.

### 3.3 Tryptophan catabolites and AhR signaling

**Figure 3:**
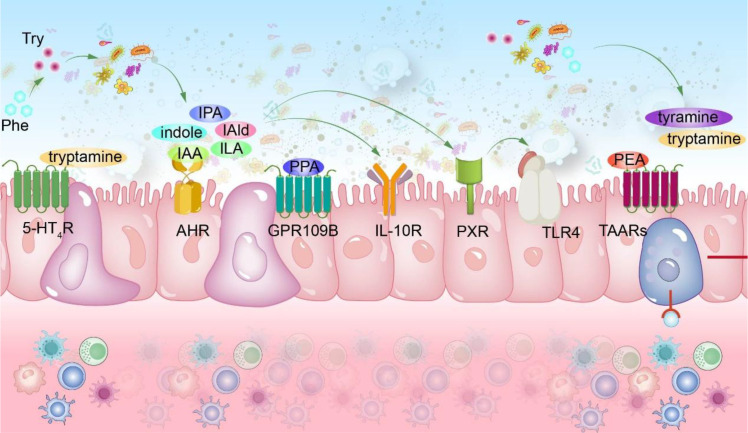
Gut microbiota–derived metabolites and their receptors. Tryptophan-derived metabolites, such as indole-3-propionic acid (IPA), indole-3-aldehyde (IAld), and indole-3-acetic acid (IAA), exert their effects mainly through the aryl hydrocarbon receptor (AHR) and the 5-hydroxytryptamine 4 receptor (5-HT4R). These metabolites are generated by gut microbes through the catabolism of tryptophan, modulate intestinal barrier function, and influence systemic physiology via gut–organ axes.

Tryptophan metabolism involves both host and microbial pathways. Host cells convert tryptophan through the kynurenine and serotonin pathways, whereas gut microbes generate indole derivatives such as indole-3-aldehyde, indole-3-acetic acid and indole-3-propionic acid that activate aryl hydrocarbon receptor (AhR), pregnane X receptor and other sensors (23). Lactobacillus reuteri-derived indole-3-aldehyde can activate AhR in ILC3s and drive IL-22 production, reinforcing mucosal barrier integrity and antifungal defense (24). Microbial indoles often support epithelial and regulatory programs, whereas host IDO-driven kynurenine can promote peripheral tolerance during acute inflammation but may become maladaptive when chronically sustained, for example by contributing to T-cell dysfunction or neurotoxic metabolite accumulation. Thus, AhR signaling is not intrinsically protective or pathogenic; its effect depends on ligand chemistry, cellular target, timing and tissue environment. Disturbed indole and kynurenine profiles in lupus, multiple sclerosis and inflammatory bowel disease place this pathway at a key intersection of microbial metabolism, host immune regulation and neuroendocrine signaling (23). **Figure 3** summarizes key gut microbiota-derived tryptophan metabolites and their receptors.

### 3.4 Other metabolite classes

Beyond SCFAs, bile acids and tryptophan derivatives, several metabolite classes broaden the microbiome-immune repertoire, although the strength of evidence varies. Polyamines such as putrescine, spermidine and spermine are produced by host and microbial pathways and can influence chromatin structure, translation, stress responses, T-cell differentiation and myeloid polarization (25, 26). Among these, spermine has mechanistic support in experimental autoimmune encephalomyelitis, where exogenous treatment reduced T-cell activation and CNS immune infiltration (113). By contrast, links between microbial vitamins, branched-chain amino acid products, hydrogen sulfide, phenolic acids and autoimmune outcomes remain more associative or context-specific in many settings (5, 20). We therefore emphasize representative mechanisms rather than treating every metabolite class as equivalently validated. The key point is that non-canonical metabolites may modulate barrier integrity, oxidative stress, inflammasome activity and immune metabolism, but their causal roles require more targeted perturbation studies.

### 3.5 Metabolic checkpoints of cross organ immunity

Across metabolite classes, microbial products can be conceptualized as metabolic checkpoints that translate intestinal ecology and nutrient availability into immune decisions in distant tissues. This checkpoint concept is more operational than the broader microbiome-immune-metabolism triangle: it identifies specific metabolite-receptor-enzyme nodes that can be measured, perturbed and therapeutically targeted. SCFAs mainly connect fiber fermentation to epigenetic regulation, GPR signaling and Treg/Breg-myeloid balance; bile acids connect cholesterol metabolism to FXR/TGR5-dependent Th17/Treg and innate programs; tryptophan catabolites connect dietary protein, microbial composition and inflammatory status to AhR-centered epithelial and lymphoid circuits; and polyamines connect growth and stress pathways to T-cell and myeloid bioenergetics (10, 23, 26–28). Together, Sections 3.1-3.4 support a unified model in which altered metabolite pools first modify barrier and immune-cell metabolic thresholds, then interact with local tissue cues to determine whether cross-organ signals resolve inflammation or amplify autoimmunity. **Table 1** summarizes representative microbiota-derived metabolites, immune targets and autoimmune contexts.

**Table 1:** Representative microbiota-derived metabolites and immune mechanisms relevant to autoimmunity.

Metabolite class	Representative metabolites	Main immune targets/pathways	Autoimmune/inflammatory context	Model/samples	Refs.
Short-chain fatty acids (SCFAs)	Acetate, propionate, butyrate	Promote colonic FOXP3^+^ regulatory T cells, support epithelial barrier integrity, limit intestinal inflammation	T cell transfer colitis, conceptually relevant to systemic autoimmunity via Treg-driven tolerance	Germ-free and specific-pathogen-free mice colonized with SCFA-producing microbiota or SCFA mixtures	(15)
Short-chain fatty acids (SCFAs)	Butyrate	Inhibits histone deacetylases and enhances Foxp3 locus acetylation, inducing colonic regulatory T cells and reducing colitis severity	Chronic intestinal inflammation that can fuel systemic immune activation	Mouse models of colitis with dietary or rectal butyrate supplementation	(112)
Secondary bile acid derivatives	3-oxo-lithocholic acid (3-oxoLCA), isoallo-lithocholic acid	3-oxoLCA directly binds RORγt and suppresses Th17 differentiation; isoalloLCA enhances mitochondrial ROS and promotes Foxp3^+^ Treg differentiation	Intestinal Th17/Treg imbalance relevant to autoimmune conditions such as IBD, arthritis and multiple sclerosis	Mouse T cell differentiation assays and *in vivo* administration of bile acid metabolites	(21)
Tryptophan-derived indole metabolites	Indole-3-aldehyde and related indole catabolites	Activate aryl hydrocarbon receptor, increase IL-22 production and reinforce mucosal barrier and antifungal defense	Control of intestinal inflammation and mucosal immune homeostasis that modulate systemic autoimmunity risk	Mouse models of fungal infection and colitis with modulation of dietary tryptophan and microbial metabolites	(24)
Polyamines	Spermine	Inhibits activation and proliferation of CD4^+^ T cells, reduces pro-inflammatory cytokine production, limits CNS immune cell infiltration	Experimental autoimmune encephalomyelitis as a model of T cell–driven CNS autoimmunity	EAE mice treated with exogenous spermine	(113)
SCFA and serotonin-pathway metabolites	Butyrate, 5-hydroxyindole-3-acetic acid (5-HIAA)	Enhance IL-10–producing regulatory B cells via aryl hydrocarbon receptor, decrease pathogenic Th17 responses and joint inflammation	Collagen-induced arthritis and human rheumatoid arthritis with reduced fecal butyrate levels	RA patient cohorts and mouse arthritis models supplemented with butyrate and 5-HIAA	(114)

## 4 Gut–organ axes: cross-organ immunity in selected autoimmune diseases

Autoimmune diseases have long been categorized by dominant target organs, yet microbiome data support a model in which gut-derived immune and metabolic cues can influence joints, kidneys, skin, lungs and the central nervous system. The evidence is heterogeneous. Human cohorts often show associations between dysbiosis, altered metabolites and disease activity, whereas stronger causal evidence comes from gnotobiotic transfer, colonization, supplementation or receptor-perturbation experiments (29–32). Therefore, the gut-organ axes below should not be read as universally reproducible microbial signatures. Rather, they illustrate how shared intestinal perturbations are filtered through local tissue microenvironments to produce distinct autoimmune phenotypes. **Table 2** highlights representative gut-organ axes and separates microbiome findings from immune or clinical relevance.

**Table 2:** Representative gut–organ axes implicated in autoimmune disease.

Gut–organ axis	Autoimmune disease	Key microbiome finding	Immune/clinical relevance	Model/samples	Refs.
Gut–joint axis	Rheumatoid arthritis	Marked expansion of intestinal Prevotella copri in new-onset, untreated RA compared with healthy controls	P. copri enrichment associated with RA susceptibility; colonization in mice increased arthritis severity, supporting a microbiota trigger for joint autoimmunity	Human cohorts with new-onset RA and controls; mouse models colonized with patient-derived P. copri	(34)
Gut–joint axis	Rheumatoid arthritis (SKG model)	Intestinal dysbiosis, including expansion of Th17-inducing taxa, increased sensitivity to arthritis; transfer of dysbiotic microbiota triggered disease	Dysbiotic microbiota activated autoreactive T cells in the intestine, leading to systemic Th17 responses and joint inflammation	SKG mice colonized with microbiota from RA patients or dysbiotic mice; human RA microbiota analyses	(36)
Gut–kidney axis	Systemic lupus erythematosus with lupus nephritis	Approximately fivefold expansion of Ruminococcus gnavus in SLE, especially in patients with high disease activity and nephritis	R. gnavus enrichment correlated with lupus disease activity and nephritis; anti-R. gnavus antibody responses linked to renal flares, implicating gut-kidney immune crosstalk	SLE patient cohorts with fecal 16S rRNA profiling and longitudinal follow-up	(115)
Gut–brain axis	Multiple sclerosis	Relatively decreased abundance of certain anti-inflammatory taxa and enrichment of species with predicted pro-inflammatory functions in relapsing-remitting MS	MS-derived microbiota altered T cell responses and promoted a more pro-inflammatory phenotype, supporting a microbiota contribution to CNS autoimmunity	RRMS patients and healthy controls; gnotobiotic mouse experiments colonized with human fecal microbiota	(116)

### 4.1 Gut–joint axis in rheumatoid arthritis and spondyloarthropathies

Rheumatoid arthritis (RA) and spondyloarthropathies (SpA) provide prototypic examples of gut-joint crosstalk (29, 33). Several fecal microbiome studies report expansion of Prevotella copri or related Prevotella taxa in new-onset or preclinical RA, together with depletion of health-associated taxa such as Bifidobacterium and butyrate-producing Clostridiales (31, 34, 35). However, P. copri findings are not fully consistent across geography, diet, disease stage and treatment exposure, and enrichment should be interpreted as one candidate pathobiont pattern rather than a universal RA biomarker. Mechanistic support is stronger in experimental systems: gnotobiotic SKG mice colonized with microbiota from patients with early RA develop more severe arthritis and increased intestinal Th17 responses (36). These data suggest that mucosal dysbiosis can promote joint inflammation by generating autoreactive or cross-reactive CD4+ T cells and IgA+ B cells that recirculate to synovium. In axial SpA and ankylosing spondylitis, altered Clostridiales and reduced SCFA-producing taxa such as Faecalibacterium prausnitzii have been reported, and butyrate-producing F. prausnitzii can ameliorate experimental SpA by restraining pathogenic Th17 and gamma-delta T-cell responses (32, 37).

### 4.2 Gut–kidney axis in lupus nephritis and other autoimmune nephropathies

In systemic lupus erythematosus (SLE), particularly lupus nephritis (LN), the gut-kidney axis is characterized by dysbiosis, barrier dysfunction and immune activation (38). Patients with SLE or LN frequently exhibit reduced microbial diversity and expansion of pathobionts such as Ruminococcus gnavus, which correlates with disease activity, renal flares and anti-dsDNA titers in some cohorts (38, 115). In humans, microbial translocation is best viewed as a permissive or amplifying process rather than definitively causative. Causal evidence is stronger in lupus-prone mouse models, where barrier disruption permits bacterial products to enter the circulation, activate TLR-NF-kappa B pathways, skew Th17 responses and aggravate renal injury (30, 55). Enterococcus gallinarum provides a mechanistic example: it can translocate from the gut to mesenteric lymph nodes and liver, induce type I interferon and ERV gp70 expression, and promote lupus-like autoimmunity in experimental systems, while immune responses to E. gallinarum-associated antigens have been detected in patients (39, 40). Similar gut-driven mechanisms are increasingly discussed in IgA nephropathy and ANCA-associated vasculitis, but their causal status remains disease- and model-dependent (41, 42).

### 4.3 Gut–skin axis in psoriasis, Behçet’s disease, and systemic sclerosis

The gut-skin axis links intestinal dysbiosis and barrier dysfunction to chronic inflammatory dermatoses such as psoriasis, Behcet’s disease and systemic sclerosis (43, 44). Psoriasis patients show altered gut microbiota composition, reduced abundance of several SCFA-producing genera, expansion of pathobionts such as Prevotella and markers of impaired intestinal tight junctions or endotoxemia (43, 45). Mechanistic work supports a model in which gut-derived metabolites and MAMPs enhance IL-23/IL-17-driven inflammation, leading to keratinocyte activation and neutrophil recruitment (46). Direct demonstration that gut-primed Th17 cells traffic to human skin lesions remains limited; in most human studies this trafficking step is inferred from shared cytokine programs, homing markers and animal data rather than directly tracked clonotypes. In Behcet’s disease, depletion of butyrate-producing Firmicutes/Clostridia and enrichment of lactic-acid-producing taxa have been linked to reduced butyrate and proinflammatory T-cell responses (47, 48). Systemic sclerosis is also associated with intestinal dysbiosis and small-intestinal bacterial overgrowth that correlate with gastrointestinal symptoms and extraintestinal manifestations, potentially supporting innate and adaptive immune activation, vasculopathy and fibrosis (44, 49).

### 4.4 Gut–lung axis in CNS and pulmonary autoimmunity (MS, ARDS, others)

**Figure 4:**
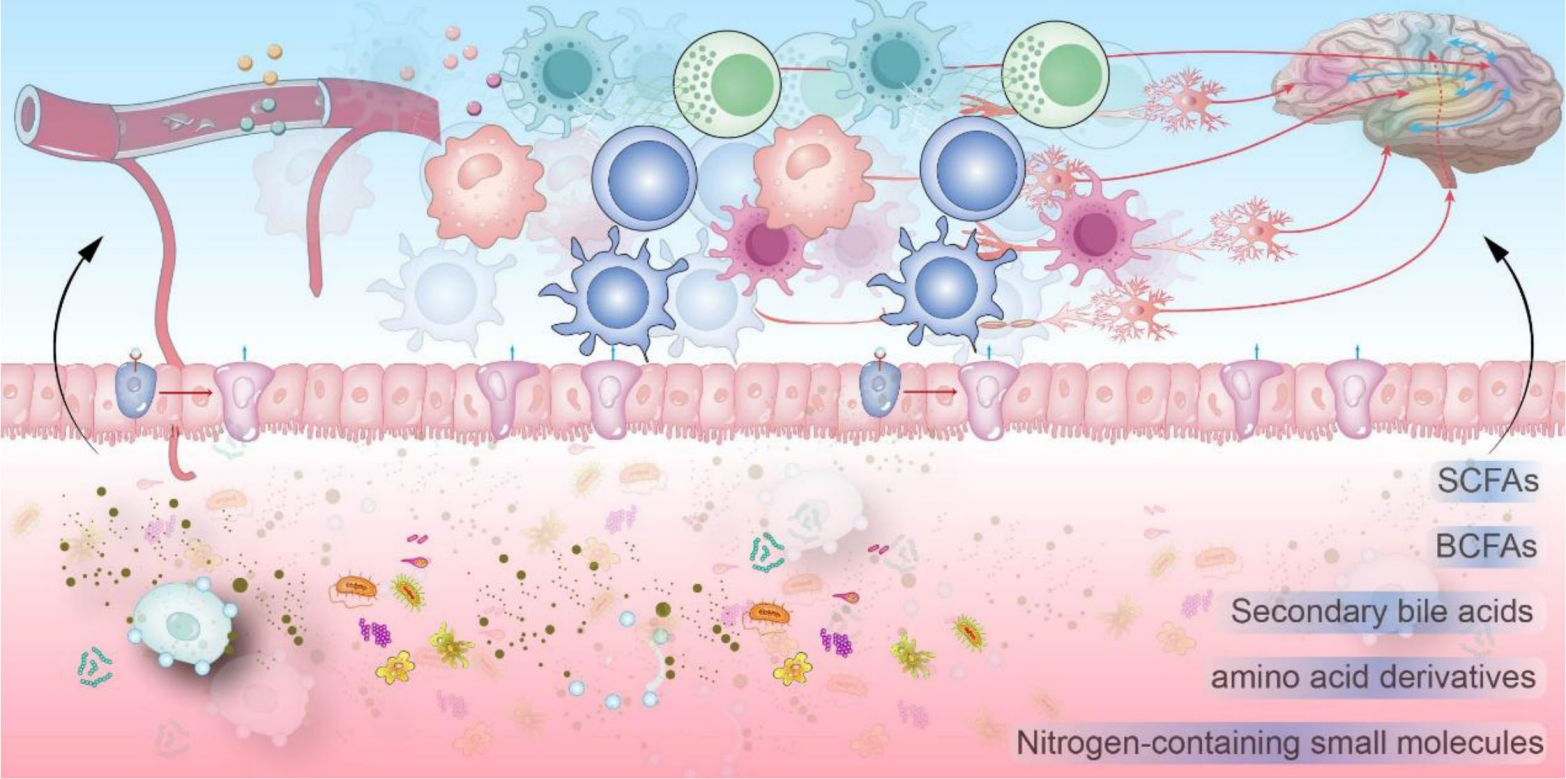
Gut microbiota–derived metabolites and the gut–brain axis.

The gut-lung and gut-brain axes integrate intestinal microbial cues with pulmonary and central nervous system (CNS) immunity (50, 51). Multiple sclerosis (MS) provides a classical autoimmune context: gut dysbiosis and reduced fecal SCFA levels have been reported in a Chinese cohort, where lower acetate, propionate and butyrate correlated with altered T-cell profiles and reduced Treg frequencies (52). Other MS cohorts also report microbial shifts involving SCFA-producing clostridial taxa and genera such as Prevotella, but the direction and magnitude of SCFA changes are not fully consistent across populations, diet and treatment status (53, 116). Experimental and translational studies strengthen causality more cautiously: propionic acid supplementation has been linked to restored Treg/Th17 balance and improved clinical and immunometabolic readouts in MS, whereas broader claims for acetate- or butyrate-mediated neuroprotection remain more model-dependent (50). **Figure 4** illustrates how gut microbiota-derived metabolites may influence the gut–brain axis by modulating intestinal barrier integrity, circulating immune mediators, microglial activation, and neuroinflammatory responses in CNS autoimmunity. ARDS is included here not as a classical autoimmune disease, but as an illustrative immune-mediated lung-injury model showing how gut-derived bacteria, metabolites and immune cells can enter or influence the lung compartment during severe systemic inflammation (51, 54). This example helps define the boundary between autoimmunity and broader cross-organ inflammatory circuits, and cautions against assuming that all gut-lung mechanisms are autoimmune-specific.

### 4.5 Common mechanisms and tissue-specific features

Across gut-joint, gut-kidney, gut-skin, gut-lung and gut-brain axes, shared mechanisms converge on a metabolite-centered hierarchy. Barrier failure increases access of microbial products and, in selected models, viable pathobionts such as Enterococcus gallinarum to systemic immune sites (39, 55). Dysregulated MAMP sensing through Toll-like receptors, NOD-like receptors and inflammasomes then promotes IL-1, IL-6, IL-17, IL-23 and TNF-dominated cytokine networks (9). Altered pools of SCFAs, bile acids and tryptophan catabolites reprogram immune metabolism and gene expression in circulating and tissue-resident cells (5). Finally, organ-specific stromal and vascular niches imprint distinct effector programs on incoming lymphocytes and myeloid cells, explaining why similar upstream gut perturbations can yield divergent phenotypes in joints, glomeruli, skin, lungs or CNS (56, 57). In simplified form, the sequence is: barrier dysfunction and dysbiosis reshape metabolite availability, metabolite changes reset immune thresholds, and tissue-specific niches determine the final autoimmune phenotype.

## 5 Sex differences in microbiome-orchestrated autoimmunity

Sex differences permeate autoimmune pathogenesis and intersect with microbiome-immune crosstalk, but the strength of evidence varies substantially by disease. Female predominance is well established for many autoimmune diseases, whereas microbiome-mediated sex bias is most directly supported in selected animal models, especially non-obese diabetic mice (58, 59, 68, 70). Human data increasingly show sex-dependent microbiome composition, hormone metabolism and immune responses, but causal links to autoimmune risk remain less mature. The microgenderome or microsexome concept is therefore useful as a heuristic framework: biological sex shapes microbial communities, microbial metabolism can influence sex hormones, and both converge on immune-cell programming (59, 62).

### 5.1 Epidemiology of sex bias in autoimmunity

Epidemiological studies estimate that approximately 80% of patients with autoimmune diseases are women, with female-to-male ratios of around 3:1 in rheumatoid arthritis and 7-9:1 in systemic lupus erythematosus and Sjogren syndrome (60). This pattern is not universal, as ankylosing spondylitis and some autoimmune liver or cholangiopathic disorders show male predominance or near-equal ratios (60, 63). X-linked immune genes, incomplete X-chromosome inactivation, TLR7 dosage and sex hormone signaling contribute to these patterns (58, 66). Estrogen generally augments humoral and type I interferon-associated pathways, whereas androgens often exert immunosuppressive effects, although both are context-dependent (64, 65). At present, direct human evidence that the microbiome causes sex bias in autoimmunity is limited; most causal support derives from animal models, with human studies providing associative evidence and biological plausibility.

### 5.2 Sex differences in gut microbiota composition and function

Human and animal studies indicate that gut microbiota composition can be sexually dimorphic, but the specific taxa that differ between males and females are highly context-dependent (59, 67). In mice, puberty, gonadal hormones, gonadectomy and hormone replacement experiments demonstrate that sex hormones shape microbial communities (68, 69). In humans, metagenomic studies report sex-dependent differences in Bacteroidetes and Firmicutes lineages, with shifts during pregnancy, menopause and aging that parallel hormonal transitions (61, 67). However, sexually dimorphic taxa are not uniformly reproducible across cohorts because diet, geography, medication exposure, age and disease state strongly modify microbiome structure. The microgenderome concept should therefore be interpreted as a bidirectional network rather than a fixed list of male- or female-enriched microbes.

### 5.3 Microbiome–sex hormone–immune triad

Studies in non-obese diabetic (NOD) mice provide proof of concept that gut microbiota can shape sex hormone levels and autoimmune risk (68, 70). In germ-free NOD mice, the usual female predominance of type 1 diabetes disappears, indicating that microbiota are required for this model-specific sex bias (70). Colonization of immature female NOD mice with adult male microbiota increases circulating testosterone, remodels microbial communities and protects females from diabetes, an effect lost when androgen signaling is blocked (68). These findings should not be generalized uncritically to all autoimmune diseases, but they demonstrate a plausible microbiome-sex hormone-immune triad. This triad connects directly to microbial metabolites: bile acids participate in hormone and lipid metabolism, SCFAs influence Treg/Th17 balance, and tryptophan catabolites regulate AhR-dependent epithelial and immune programs. Sex-dependent differences in these metabolite pools may therefore help translate microbial community differences into divergent immune thresholds.

### 5.4 Implications for metabolite-focused and microbiome-based therapies

Recognition of sex differences in the microbiome and immune system has important implications for metabolite-focused and microbiome-targeted interventions in autoimmunity (71). Preclinical studies show that similar microbial consortia or metabolites can produce distinct immunological effects in males and females, partly through androgen levels and effector/regulatory T-cell balance (68). However, most current clinical trials of prebiotics, probiotics, live biotherapeutics and FMT are not powered for sex-stratified efficacy or safety analyses, and few incorporate hormonal status, menstrual stage or menopausal status as design variables (59, 88). Practical challenges include larger sample-size requirements, variability in hormone exposure, medication confounding and the need to avoid overfitting subgroup analyses. Even so, sex-aware design should be considered essential for microbiome-based autoimmunity trials because an intervention validated in one sex may be neutral, less durable or even mechanistically distinct in the other.

## 6 Technological advances to map cross-organ microbiome–immune networks

Rapid technological progress is transforming the study of microbiome-orchestrated cross-organ immunity from descriptive association to mechanistic, system-level mapping. The revised section combines microbiome-metabolome profiling with single-cell and spatial immune profiling because these approaches are complementary components of the same analytical pipeline. We use immune profiling or immune landscape to describe high-dimensional characterization of immune cells, cytokines, receptors and repertoires. These tools have already advanced autoimmune research by linking microbial functions and metabolites to immune endotypes in rheumatoid arthritis, systemic lupus erythematosus and multiple sclerosis, although many applications remain proof-of-concept. Important limitations include batch effects, tissue-sampling bias, high cost, computational reproducibility and the difficulty of proving causal cross-organ trafficking in humans (72–76).

### 6.1 Integrated microbiome-metabolome-immune and spatial profiling

Integrated microbiome-metabolome-immune profiling captures, within the same individuals, gut microbial composition and function, metabolite pools and immune-cell states (75). Amplicon or shotgun metagenomics define taxonomic and functional shifts, LC-MS- or NMR-based metabolomics quantify SCFAs, bile acids, tryptophan derivatives and lipids, and multiparameter flow cytometry, mass cytometry, TCR/BCR sequencing and cytokine profiling link these molecules to Th17, Tfh, Treg or autoreactive B-cell programs (75, 76). In rheumatoid arthritis, systemic lupus erythematosus and multiple sclerosis, metabolomics has revealed coordinated changes in amino acids, lipids and energy intermediates that correlate with disease activity and treatment response, but most signatures are not yet prospectively validated for clinical use (76). Examples from myalgic encephalomyelitis/chronic fatigue syndrome are included only as methodological proof-of-concept for deep stool metagenomics, plasma metabolomics and clinical phenotyping; they should not be interpreted as direct autoimmune evidence (73). Single-cell RNA sequencing, CITE-seq, spatial transcriptomics and spatial proteomics add cellular and anatomical resolution by mapping microbiota-sensitive immune niches and tissue architecture (72, 77–79). In humans, direct cross-organ tracing of microbiome-educated immune cells from gut to target tissue remains largely aspirational because matched gut, blood and inflamed-organ samples are difficult to obtain and integrate. Future autoimmune studies should therefore pair these technologies with targeted validation in experimental models and carefully harmonized sampling protocols.

### 6.2 Cross-tissue atlases and organ-on-chip models

Cross-tissue atlases and organ-on-chip systems provide complementary frameworks to test candidate microbiome-immune mechanisms (72, 80). A multi-organ atlas comparing germ-free and specific-pathogen-free mice used spatial transcriptomics, single-cell RNA-seq and targeted bile acid metabolomics across intestinal, lymphoid and metabolic organs, showing that microbiota absence reprograms B-cell, myeloid and NK/T-cell compartments and disrupts bile acid and lipid homeostasis in a coordinated but tissue-specific manner (72). Such atlases provide mechanistic reference maps, but they do not replace disease-specific validation in autoimmune models or patient tissues. Microfluidic gut-on-chip models enable controlled interrogation of human intestinal epithelium co-cultured with microbes under physiologic flow and mechanical strain (81). Multi-organ chips that connect gut modules to liver, vascular or brain compartments may help test how microbial products and inflammatory mediators propagate across interfaces, although current systems simplify vascular, neural and immune complexity (82).

### 6.3 Computational modeling and causal inference

Computational approaches are essential for integrating microbial taxa, functional pathways, host metabolomics, immune phenotypes and clinical outcomes into candidate microbiome-metabolite-immune modules (83, 84). Network analyses can prioritize co-varying microbial functions and metabolites, while machine-learning models can classify disease or treatment-response endotypes, although overfitting and lack of external validation remain major risks (73, 83). Mendelian randomization (MR) has strengthened causal inference by using host genetic variants as instruments for microbial taxa or functions in studies of autoimmune and immune-mediated diseases, but conclusions are limited by weak instruments, horizontal pleiotropy, population specificity and the fact that most microbial traits are measured at coarse taxonomic resolution (8, 106). Consequently, computational predictions should be treated as hypothesis-generating unless supported by experimental perturbation in organ-on-chip platforms, germ-free or gnotobiotic models, metabolite supplementation studies or prospective interventional cohorts. Combining causal-inference tools with experimental validation will be necessary to move from correlation-rich maps to mechanism-based microbiome and metabolite interventions.

## 7 From metabolites to therapeutic targets: translational opportunities

Microbiome-derived metabolites provide a tractable interface between environmental inputs, microbial community structure and host immune circuits, but therapeutic evidence remains uneven (5, 86, 88). Dietary interventions, prebiotics, probiotics, synbiotics, fecal microbiota transplantation, engineered consortia and metabolite-receptor modulators span a continuum from ecosystem-level manipulation to molecular targeting (85, 86, 88). In this section we distinguish preclinical mechanisms, exploratory case reports, small pilot trials and larger randomized evidence to avoid overstating clinical readiness. Translational challenges include inter-individual variability, limited durability, colonization resistance, regulatory complexity, long-term safety and the need for biomarker- and sex-aware patient stratification. **Table 3** summarizes representative microbiome- and metabolite-targeted interventions in autoimmune and inflammatory arthritis, highlighting how dietary fiber, probiotics, synbiotics, fecal microbiota transplantation, and defined metabolite supplementation can reshape immune and clinical outcomes with different levels of evidence.

**Table 3:** Microbiome- and metabolite-targeted interventions in autoimmune and inflammatory arthritis.

Intervention type	Disease/model	Key microbiome or metabolite change	Clinical/immunologic outcome	Study design	Refs.
Short-term high-fiber supplementation	Arthritis patients including RA	Oat-bran–based high-fiber diet increased circulating SCFA levels and shifted gut microbiota composition toward more saccharolytic taxa	Reduced circulating inflammatory mediators and improvement of some patient-reported outcomes, supporting dietary fiber as an adjunct anti-inflammatory strategy	Open-label interventional trial in patients with chronic inflammatory arthritis	(107)
Probiotic Lactobacillus casei 01	Rheumatoid arthritis	Daily L. casei 01 supplementation modestly modified gut microbiota and systemic cytokine profile	Decrease in serum IL-6 and TNF-α and improvement in Disease Activity Score in some patients, suggesting probiotic-mediated immune modulation	Randomized, double-blind, placebo-controlled clinical trial in RA	(117)
Synbiotic (probiotic plus prebiotic)	Rheumatoid arthritis	Synbiotic supplementation was designed to modulate gut microbiota, but the trial did not directly quantify microbiome composition or metabolite output	No significant between-group differences in TJC28, SJC28, VAS, ESR, CRP or DAS28 after 3 months; response rates were similar between synbiotic and placebo groups	Randomized, placebo-controlled adjuvant trial in RA patients receiving routine antirheumatic therapy	(118)
Fecal microbiota transplantation (FMT)	Refractory rheumatoid arthritis	Transfer of stool from a healthy donor reshaped gut microbiota composition in a refractory RA patient	Marked and sustained clinical improvement in joint pain and function, allowing reduction of conventional medications, highlighting FMT as a potential rescue therapy	Single-patient case report with longitudinal follow-up	(94)
Microbiota-derived metabolite supplementation (butyrate and 5-HIAA)	Collagen-induced arthritis and RA	Supplementation with butyrate and serotonin-pathway metabolite 5-HIAA restored levels of IL-10–producing regulatory B cells and partially corrected SCFA deficiency	Attenuated arthritis severity in mice and associated with reduced inflammatory cytokines in RA, supporting metabolite-based strategies to reinforce tolerance	Mechanistic preclinical study plus analyses of stool and B cells from RA patient cohorts	(114)

### 7.1 Dietary interventions and metabolite-directed nutrition

Dietary fiber and complex carbohydrates are primary substrates for colonic SCFA production, and controlled feeding studies in arthritis show that targeted fiber enrichment can increase circulating acetate, propionate and butyrate. In a pilot trial in rheumatoid arthritis and osteoarthritis, a short-term high-fiber intervention increased systemic SCFA levels, shifted the fecal microbiota toward saccharolytic taxa and was associated with reduced inflammatory mediators and improved patient-reported outcomes (107). These results support biological plausibility but remain preliminary and require replication in larger, controlled cohorts. Mechanistically, SCFAs signal through GPR41, GPR43 and GPR109A and inhibit histone deacetylases, promoting Treg differentiation, limiting Th17 polarization and enhancing barrier integrity in preclinical models (10, 11). Observational associations between Mediterranean-style diets, higher SCFA levels and lower inflammatory markers should be distinguished from interventional evidence, because diet patterns also capture lifestyle, medication and socioeconomic factors. Metabolite-directed nutrition in autoimmunity should therefore be viewed as an adjunct strategy requiring standardized fiber formulations, objective metabolite readouts and clinical endpoints rather than as a broadly established therapy.

Prebiotic fibers such as inulin, resistant starch and fructo-oligosaccharides can selectively expand SCFA-producing taxa and suppress pathobiont expansion, but clinical effects depend on baseline diet, microbial colonization capacity and host treatment context. Observational links between plant-rich or Mediterranean-style diets, higher microbial diversity and reduced inflammatory markers are useful for hypothesis generation but cannot prove that specific metabolites drive clinical benefit. In contrast, interventional trials can directly test whether changing fiber substrates modifies SCFA pools, immune phenotypes and disease activity. Future nutrition studies should therefore combine controlled dietary exposure, objective adherence metrics, stool and plasma metabolomics, and prespecified inflammatory endpoints.

### 7.2 Probiotics, next-generation probiotics, and postbiotics

Conventional probiotics, typically Lactobacillus and Bifidobacterium species, can enhance barrier function, compete with pathobionts and modulate mucosal cytokines, but their effects in systemic autoimmunity are modest and heterogeneous (87). A meta-analysis of 80 randomized trials across autoimmune and rheumatic diseases reported small-to-moderate improvements in disease activity indices and inflammatory markers with generally favorable safety, but strain identity, dose, duration, background therapy and endpoints varied widely (88). In SLE, a randomized trial of Bifidobacterium animalis subsp. lactis with inulin improved dysbiosis, increased circulating Tregs and reduced disease activity scores, suggesting that carefully selected synbiotics may influence metabolite-immune networks (89). However, colonization efficiency, persistence after discontinuation and long-term durability remain uncertain. Next-generation probiotics based on Faecalibacterium prausnitzii, Akkermansia muciniphila or selected Clostridia clusters and postbiotic metabolite formulations are promising but largely developmental outside selected indications (90–92). Thus, established clinical benefit should be separated from emerging strategies aimed at rationally engineering metabolite landscapes.

### 7.3 Fecal microbiota transplantation and microbial ecosystem engineering

FMT provides a high-intensity intervention that replaces a microbial ecosystem and its metabolite output, with established efficacy in recurrent Clostridioides difficile infection but much less certain efficacy in autoimmune diseases (93). The reported RA case with marked improvement after serial FMT should be framed as exploratory rather than definitive, because single-patient responses cannot exclude placebo effects, regression to the mean or concurrent medication influences (94). Systematic reviews across autoimmune and autoinflammatory indications suggest potential benefit and acceptable short-term safety, but sample sizes are modest, protocols are heterogeneous and evidence is strongest in intestinal immune-mediated disease rather than systemic autoimmunity beyond IBD (95). Engineered consortia attempt to retain functional attributes such as SCFA production, bile-acid transformation and tryptophan catabolism while reducing risks associated with whole-stool transfer (96, 97). Their development raises regulatory and safety issues, including donor screening, manufacturing consistency, antimicrobial resistance, horizontal gene transfer and long-term ecological effects (98).

### 7.4 Pharmacological targeting of metabolite pathways

Pharmacologic targeting of metabolite pathways offers a more defined alternative to ecosystem-level intervention, but most targets remain preclinical or early translational in autoimmunity. FXR and TGR5 modulators can influence intestinal barrier integrity, innate immune activation and hepatic-intestinal crosstalk, yet their autoimmune applications are largely inferred from experimental and related inflammatory disease contexts (27, 28). AhR ligands illustrate the distinction between direct metabolite-receptor targeting and broader immunomodulators with microbiome-linked pathways; for example, laquinimod requires AhR and c-Jun to reduce CNS inflammatory infiltrates in experimental autoimmune encephalomyelitis, but it is not simply a microbial metabolite replacement strategy (99). SCFA receptor agonists and histone deacetylase inhibitors mimic selected SCFA effects, including Treg induction and reduced effector cytokine production, but tissue specificity, dose, receptor promiscuity and off-target epigenetic effects are major translational considerations (100, 101). Future development should prioritize tissue-selective modulators that dampen pathogenic immune circuits without disrupting homeostatic metabolite signaling.

### 7.5 Patient stratification and biomarker-driven clinical trials

A recurring theme across microbiome-metabolite interventions is heterogeneity of response, suggesting that baseline microbiome state, metabolome, host genetics, sex, diet and background therapy shape outcomes (88). Pretreatment microbiome composition has been associated with later response or non-response to methotrexate or TNF antagonists in rheumatoid arthritis and inflammatory bowel disease, but prospective validation of microbiome-based stratification remains limited (102, 103). Metabolomic profiling adds functional resolution by quantifying SCFAs, bile acids and aromatic amino-acid metabolites that track with inflammation and treatment response (104). Practical barriers include sample handling, assay standardization, cost, turnaround time, regulatory qualification of biomarkers and integration with clinical decision-making. A concise precision strategy would use baseline microbiome-metabolome profiles to select a metabolite-directed intervention, then use serial metabolite and immune readouts as pharmacodynamic markers to confirm target engagement.

## 8 Challenges, knowledge gaps, and future directions

Accumulating data support a central role for the microbiome and its metabolites in autoimmunity, yet the gaps identified below map directly onto the preceding framework. Metabolite-centered mechanisms require stronger causal validation, cross-organ models require better tissue sampling and trajectory analysis, and therapeutic translation requires safety, regulatory and biomarker standards. Current evidence remains dominated by cross-sectional association studies with heterogeneous cohorts, variable sampling protocols and incomplete control of diet, medication and comorbidities. A coordinated roadmap should integrate longitudinal human cohorts, mechanistic experimental systems and rigorous computational inference to test whether defined microbiome-metabolite modules can alter autoimmune trajectories rather than merely correlate with them.

### 8.1 Causality vs association

Most microbiome-autoimmunity studies still describe compositional differences between patients and controls, which are vulnerable to confounding and reverse causation (105). Few autoimmune cohorts currently meet the ideal standard of dense longitudinal sampling before disease onset, during flare, remission and treatment transition with harmonized diet, medication and multi-organ immune readouts. Mendelian randomization analyses support putative causal links between specific gut microbes and autoimmune diseases such as rheumatoid arthritis, multiple sclerosis and systemic lupus erythematosus, but effect sizes are modest and instruments are often weak (8, 106). Horizontal pleiotropy, population stratification and taxonomic imprecision further limit interpretation. Controlled dietary, probiotic or metabolite-intervention trials with prespecified immune endpoints provide a complementary path beyond correlation (88, 107). A priority is to combine microbial or metabolite perturbation with deep immunophenotyping and target-organ readouts to test whether manipulating defined microbial functions can alter flares, progression or treatment responses in a tissue-specific manner (76, 108).

### 8.2 Inter individual variability and context dependence

Marked inter individual variability in microbiome composition and function complicates the definition of disease associated signatures and therapeutic targets. Large population studies demonstrate that geography, diet, lifestyle, antibiotics, and other medications explain a substantial fraction of microbiome variance, often exceeding the contribution of single host genetic loci (109). Even among “healthy” individuals, there is a wide continuum of taxonomic and metabolic configurations, which challenges the use of simple dysbiosis labels and undermines the notion of a single healthy reference state (110). Autoimmune patients add further layers of heterogeneity, including age, sex, disease duration, organ involvement, background immunosuppression, and comorbid metabolic or infectious conditions. These contextual factors modulate metabolite production, barrier function, and immune tone, so that the same microbial pathway may be protective in one setting and pathogenic in another. Future work should adopt stratified designs that pre specify clinically relevant subgroups, use harmonized sampling and analytical pipelines, and explicitly model interactions between microbiome features, host variables, and treatments, rather than adjusting them away as mere confounders (108). 

### 8.3 Model and measurement limitations

Germ free and gnotobiotic mice, antibiotic depletion, and fecal transplantation models have been invaluable for demonstrating that microbiota can shape immune development and autoimmune susceptibility, but they are far from the complexity of human exposures. Germ free animals exhibit profound immune, metabolic, and neuroendocrine abnormalities that may exaggerate effect sizes and obscure nuances relevant to conventional hosts (108). Specific pathogen free facilities, standardized chow, and restricted genetic backgrounds limit the diversity of microbes and environmental stimuli, which may not capture the environmental, dietary, medication-related and geographic variability seen in human populations (109). On the measurement side, differences in sampling site, storage, DNA extraction, sequencing platform, metabolite derivatization, and bioinformatic pipelines introduce substantial batch effects that complicate cross study comparison. Short chain fatty acids, bile acids, and tryptophan catabolites are highly labile and sensitive to preanalytical handling, yet protocols are often poorly standardized. There is a need for interoperable reference materials, cross laboratory proficiency testing, and reporting standards that cover both microbiome and metabolome layers, coupled to functional readouts such as ex vivo immune assays or *in vivo* imaging to reduce the gap between descriptive omics and mechanistic insight.

### 8.4 Safety, ethics, and regulatory considerations

Microbiome targeted interventions raise specific safety and regulatory challenges that are particularly salient in chronic autoimmune diseases. Fecal microbiota transplantation is highly effective for recurrent Clostridioides difficile infection, but its long term safety in non-infectious indications is uncertain, and cases of pathogen transmission have prompted stringent donor screening requirements (111). For autoimmune indications such as multiple sclerosis or type 1 diabetes, available trials are small, often open label, and rarely powered to detect delayed adverse events, malignant transformation, or off target immune activation. Probiotic and next generation live biotherapeutic products blur the boundary between foods and drugs and may require new regulatory categories, particularly when engineered strains with synthetic genetic circuits are used (108). Ethical considerations include informed consent for complex multi donor products, equitable access to personalized microbiome therapies, and stewardship of microbiome data as a sensitive biomarker of identity and disease risk. Regulatory frameworks will need to consider manufacturing consistency, horizontal gene transfer, antimicrobial resistance, and ecological impacts of releasing engineered consortia, while also providing flexible pathways for adaptive trial designs in rapidly evolving fields (110).

### 8.5 Future roadmap

A future research agenda for microbiome-orchestrated autoimmunity should move from descriptive taxa lists toward mechanistic, cross-organ networks integrating microbial genes, metabolites, immune circuits and tissue microenvironments. Conceptual frameworks treating the microbiome as an adaptive genome interacting with the host innate genome provide a starting point for systems-level models (108). Longitudinal cohorts with harmonized stool, blood and affected-tissue multi-omics should be paired with validation in refined animal, organoid and organ-on-chip systems (108). A concrete example would be a rheumatoid arthritis trial enrolling patients with low fecal butyrate and reduced butyrate-producing taxa, randomizing them to a targeted fiber or butyrate-delivery intervention, and using serial SCFA levels, Treg/Th17 balance, synovial inflammation and disease activity as integrated endpoints. Similar metabolite-module trials could be designed for bile-acid pathways in lupus nephritis or AhR-active indoles in psoriasis or multiple sclerosis. Iterative cycles of computational modeling, experimental perturbation and early-phase clinical trials should be embedded within learning health systems that update benefit-risk estimates over time.

## 9 Conclusions

Autoimmune diseases are increasingly understood as systemic disorders in which immune activation at barrier sites, particularly the gut, shapes pathology in distant organs. Across rheumatoid arthritis, spondyloarthropathies, lupus nephritis, psoriasis, multiple sclerosis and other immune mediated conditions, convergent evidence points to microbial metabolites as central mediators of this cross organ communication. Short chain fatty acids, bile acid derivatives, tryptophan catabolites, polyamines and other small molecules form a biochemical interface between diet, microbiota and host tissues. By acting on G protein coupled receptors, nuclear receptors, epigenetic enzymes and metabolic pathways in immune and non-immune cells, these metabolites calibrate the balance between effector and regulatory programs that drive or restrain autoimmunity in joints, kidneys, skin, lungs and the central nervous system.

This conceptual shift reframes microbial metabolites as druggable nodes that connect three key dimensions of autoimmune pathogenesis. The microbiota provides the enzymatic machinery that generates and transforms metabolite pools. The immune system interprets these signals through context dependent receptor engagement and intracellular metabolic rewiring. Tissue specific microenvironments imprint additional layers of regulation on infiltrating lymphocytes and myeloid cells, shaping organ restricted clinical phenotypes. Focusing on metabolite pathways offers a practical handle on this complexity, since small molecules can be modulated by diet, targeted microbial interventions or pharmacologic agents, and can be measured longitudinally as biomarkers of pathway engagement.

Translationally, this view supports a spectrum of interventions that range from ecosystem level strategies to precise molecular targeting. Fiber enriched and metabolite directed diets, probiotics and next generation probiotics, postbiotic formulations, fecal microbiota transplantation and engineered consortia collectively aim to restore health associated metabolite landscapes and gut barrier function. In parallel, small molecules that agonize or modulate FXR, TGR5, aryl hydrocarbon receptor or SCFA receptors, or that inhibit detrimental metabolic pathways, offer more direct entry points into metabolite signaling cascades. Evidence from early clinical studies and preclinical models indicates that these approaches can influence disease activity and immune signatures, but heterogeneity of response and limited mechanistic resolution currently constrain rational deployment.

Bridging this gap requires integration of advanced technologies and study designs. Multi-layer profiling of microbiome, metabolome and immune landscapes in longitudinal cohorts can define stable and dynamic components of the microbiome-metabolite-immune network across disease stages, therapies, sex and environmental contexts. Single-cell and spatial multi-omics across gut, blood and target organs, together with cross-tissue atlases in germ-free and specific-pathogen-free models, can map microbiota-dependent immune circuits in anatomical detail. Organ-on-chip systems enable controlled interrogation of gut-organ axes, while computational modeling, including network analysis and Mendelian randomization, can prioritize causal metabolite-immune-disease links for experimental testing.

Future progress will depend on embedding these insights in precision clinical trials that treat microbiome and metabolome features as integral variables rather than background noise. Sex, age, genetics, diet and medication use should be recognized as core determinants of microbiome-dependent immunity and incorporated into trial design and analysis. Microbiome and metabolite signatures have the potential to serve as predictive, prognostic and pharmacodynamic biomarkers that guide selection and monitoring of metabolite-focused interventions. Ultimately, microbial metabolites should be viewed as actionable cross-organ immune checkpoints: they connect intestinal ecology to tissue-specific autoimmunity and provide measurable, targetable nodes for moving from broad immunosuppression toward rational modulation of systemic and organ-level immune circuits.
